# Dual biologic therapy for the treatment of rheumatic diseases and asthma: a case series

**DOI:** 10.1093/rap/rkad018

**Published:** 2023-02-03

**Authors:** Mariam Malik, Bryony Jones, Emma Williams, Ramesh Kurukulaaratchy, Chris Holroyd, Alice Mason

**Affiliations:** Rheumatology Department, University Hospital Southampton, Southampton, UK; Rheumatology Department, University Hospital Southampton, Southampton, UK; Rheumatology Department, Royal Hampshire County Hospital, Winchester, UK; Department of Respiratory Medicine, University Hospital Southampton, Southampton, UK; Rheumatology Department, University Hospital Southampton, Southampton, UK; Rheumatology Department, University Hospital Southampton, Southampton, UK

**Keywords:** rheumatic disease, asthma, immunosuppressants, biological therapies, infections and arthritis, quality of life, etanercept, mepolizumab, infliximab, omalizumab

## Abstract

**Objective:**

Combination biological therapies are being considered increasingly for patients with multiple co-morbidities requiring biologics. There are limited data available on this approach, and concerns remain about the possible risk of adverse events, particularly infection.

**Methods:**

We present three patients on dual biologics for rheumatic disease and asthma. The biologic combinations used were etanercept and mepolizumab, infliximab and omalizumab, and etanercept and omalizumab. The time on combination biologic therapies ranged from 24 to 36 months. Patients were monitored for any serious adverse events.

**Results:**

All three patients were able to tolerate combined biologic therapies, with no serious adverse events. All three patients gained improvement in their rheumatic and asthma disease control, with reduction in disease activity scores and reduction in steroid usage.

**Conclusion:**

The decision to start dual biologic therapy should be considered carefully, on a case-by-case basis. The number of patients who are on combination biological therapy is small, and data are sparse. Real-world data are needed to examine the long-term benefits and risks of different forms of combination biologic therapies.

Key messages

- Combination biological therapies are being considered increasingly for patients who present with multiple co-morbidities.
- Data presented here suggest that combination biologics can be used safely, without serious adverse events.
- The decision to start dual biologic therapy should be considered carefully, on a case-by-case basis.

## Introduction

Combination biological therapies are being considered increasingly for the treatment of patients who present with multiple co-morbidities requiring biological treatment. There are limited data available regarding this, and concerns remain about the risk of adverse events, particularly infections, in addition to potential drug interactions in patients receiving more than one biological therapy [1]. We present a case series of three patients who have benefited from dual biologic therapy for rheumatic disease and asthma.

Literature on dual biologic therapy is limited, owing to the infrequency of this clinical approach. There are significant clinical reservations in taking this approach owing to the potential risk of serious infection from immunosuppression [1]. In addition, the wide variety of biological therapies and their potential combinations makes consistent comparison of outcomes difficult, and structured clinical trials are infrequent.

Development of newer agents targeting different pathways has made it possible to use combination therapy in selected patients. Simultaneous use of more than one biological agent has been used in two main scenarios: patients with severe inflammatory disease unresponsive to a single biological agent, or patients with co-existing severe medical conditions, such as Inflammatory Bowel Disease [2–4], asthma [5, 6] and psoriasis [7]. There is limited real world evidence from case-based literature on combining biological therapies across different clinical specialties.

## Methods

We present three cases illustrating the outcome of combination therapy for severe asthma and inflammatory arthritis. Formal ethics approval was not required for this work because only anonymized patient information was included and no change to patients’ standard care was required in order to be included in the case series. All patients gave written informed consent to the use of their data. A summary of the clinical presentations and therapies can be found in Table 1.

**Table 1.** Summary of key points of each case

Age (years)	Diagnosis	Medications (biologics)	Serious adverse events	Rheumatic disease control
56	Seronegative RA Asthma	Etanercept, 2020 Mepolizumab, 2018	Nil	Continues prednisolone 11 mg with no evidence of active synovitis on US, although it has been difficult to reduce prednisolone further
35	Crohn’s-associated arthritis Crohn’s disease Asthma	Infliximab, 2008 Omalizumab, 2019	Nil	Remission
62	Seropositive RA Asthma	Omalizumab, 2019 Etanercept, 2018	Nil	Remission

Etanercept and infliximab are anti-TNF-α agents licensed for the treatment of moderate or severe RA [8]. Omalizumab is an anti-IgE biologic used for the treatment of atopic asthma [9]. Mepolizumab is an anti-IL-5 agent used for the treatment of severe eosinophilic asthma.

We have defined serious adverse events in accordance with the US Food and Drug Administration definition [10]. A serious adverse event is an undesirable experience associated with the use of a medical product, which is life threatening, can result in death, hospitalization, disability, permanent damage or congenital anomaly. This also includes other serious events, such as allergic bronchospasm, serious blood dyscrasias or seizures. We have also included transaminitis and infections requiring antibiotics. Information was gathered by reviewing medical notes at the tertiary centre, the local hospital, from general practitioner records and by contacting patients.

## Case one

Case one is a 56-year-old woman with severe eosinophilic asthma diagnosed in the late 1990s and seronegative RA diagnosed in 2019. Her asthma was previously treated with bronchodilator and steroid inhalers, montelukast and daily maintenance prednisolone. Mepolizumab was commenced in 2018, and since then she has reported no exacerbations of her asthma. Mid-2022, she was reviewed by the asthma service and was tolerating mepolizumab well, was no longer on montelukast or the steroid inhaler, and had not had to use her salbutamol inhaler for ∼7 months.

For RA, she was initially treated with sulphasalazine (SSZ), but this was not tolerated and was stopped. She commenced methotrexate (MTX) 20 mg s.c. once weekly along with prednisolone in early 2020, but after 6 months remained symptomatic, with evidence of active synovitis on musculoskeletal ultrasound (US), despite being on prednisolone 25 mg daily. At this time, her disease activity score (DAS28) was 6.25.

Etanercept was then commenced alongside MTX and prednisolone. Twenty-four months after commencing etanercept, she continues etanercept and has reduced her prednisolone to 11 mg daily. Her DAS is 2.27. It has been difficult to reduce her prednisolone further thus far owing to ongoing joint pain, although there is no synovitis on US. She is being encouraged to reduce the corticosteroids (CS) dose gradually. She has been on dual therapy for 24 months and has not had any recorded serious adverse events.

## Case two

Case two is a 35-year-old man, known to have asthma since childhood, requiring multiple hospital admissions and an intensive care unit admission in 2002. He has previously been on a combination of inhalers, aminophylline and montelukast, but still required frequent courses of oral CSs. He was diagnosed with Crohn’s disease in 2006 and was subsequently diagnosed with Crohn’s-associated arthritis, for which he has been on azathioprine (AZA) and infliximab since 2008. In April 2019 he was started on omalizumab for severe asthma that was difficult to control. On follow-up, he reported significant improvement in asthma symptoms, with no further need for oral CSs, and he no longer requires nebulized therapy. He continues infliximab at the same dose and omalizumab and has achieved good control of his Crohn’s disease and associated arthritis. He has not experienced any serious adverse effects after 36 months of combination therapy.

## Case three

Case three is a 62-year-old woman who was diagnosed with atopic eosinophilic adult-onset asthma in the late 1980s and seropositive RA in 2012. Prior therapy for asthma included a combination of bronchodilator and steroid inhalers. She was referred to the severe asthma service in 2018, and omalizumab was commenced in June 2019. Since starting this, her asthma has been well controlled, supported by clinical examination and ACQ-6 score (asthma control questionnaire). Initially, her RA was treated with MTX and hydroxychloroquine (HCQ). However, despite this combination she had evidence of ongoing joint inflammation, and in 2018 she was started on etanercept. She has continued both biologics for 36 months with good control of asthma and RA, without any serious adverse events, and has not required any steroid therapy. This was confirmed by reviewing the omalizumab record sheet.

## Results

In these three cases, dual biologic therapy has been used to good effect, with no infection or other serious adverse events. This has also enabled patients to reduce oral CS dependence significantly, with the added benefit of minimizing CS-related adverse effects.

## Discussion

The decision to start a patient on dual biologic therapy should be considered carefully, on a case-by-case basis, only when conventional treatment options have failed and in the context of life-threatening or significantly disabling disease. The main concerns regarding the use of dual biologic therapy centre around safety, especially a theoretical risk of serious infections [1]; no infections were seen in this small case series. This potential risk must be balanced carefully against the potential long-term harm from ongoing severe disease and the well-documented risks associated with other adjuvant therapies, specifically CSs, which would be the most likely alternative treatment option in this group of difficult-to-treat patients. The number of patients requiring combination biological therapies is a small but important group. Data are sparse, because they are generally excluded from clinical trials. Thus, real-world data are important and needed to examine the long-term benefits and risks of different forms of combination biologic therapies across different disease areas.

## Data availability

The data underlying this article cannot be shared publicly owing to ethical/privacy reasons.
